# The role and regulatory mechanisms of the TCA cycle in early embryonic development

**DOI:** 10.3389/fcell.2025.1662431

**Published:** 2025-09-19

**Authors:** Yipan Lai, Xiurong Gao, Liwen Zhao, Jin Liu, Chao Gao, Qingfu Yan, Yangneng Zeng, Zibing Liao, Jianing Zhong

**Affiliations:** ^1^ School of Basic Medical Sciences, Gannan Medical University, Ganzhou, China; ^2^ Key Laboratory of Prevention and Treatment of Cardiovascular and Cerebrovascular Diseases of Ministry of Education, Gannan Medical University, Ganzhou, China

**Keywords:** TCA cycle, embryonic development, zygotic genome activation, energymetabolism, nuclear localization, epigenetic modification

## Abstract

The tricarboxylic acid cycle (TCA cycle) serves as a critical metabolic hub in embryonic development. Its dynamic reprogramming not only coordinates energy supply and biosynthesis but also profoundly influences cell fate decisions through the metabolic-epigenetic coupling mechanism. This review systematically explores the TCA cycle central role in driving the adaptive metabolic changes of embryos, such as mitochondrial maturation and lineage differentiation, and precisely regulating the timing of zygotic genome activation (ZGA). It highlights how the nuclear translocation of key enzymes in the TCA cycle creates a nuclear metabolic microenvironment, which directly regulates histone modifications (acetylation, methylation) and DNA demethylation through intermediate products like Ac-CoA and α-ketoglutarate (α-KG), thereby achieving epigenetic remodeling. Additionally, the review emphasizes the pathological mechanisms by which mitochondrial dysfunction (such as insufficient ATP synthesis, abnormal metabolite accumulation, and oxidative stress imbalance) leads to developmental arrest through epigenetic disorders and DNA damage.

## 1 Introduction

Embryonic development is a highly coordinated and precise process that involves the establishment of pluripotency and lineage-specific differentiation. This process faces three core challenges: the continuous demand for energy supply, large-scale epigenetic remodeling, and the integration of spatiotemporal-specific signals (Tarazona and Pourquié, 2020). Traditionally, the TCA cycle has been seen as the core of the mitochondrial energy factory, primarily responsible for producing ATP and reducing power (NADH/FADH2), and meeting the rapid energy demands of embryonic division through oxidative phosphorylation (OXPHOS).

However, recent studies have challenged this perception, revealing that the TCA cycle plays a much broader role in embryonic development than just energy metabolism. It has evolved into a multifaceted regulatory hub, directly engaging in epigenetic modifications, gene expression regulation, and cell fate decisions through its key intermediates (such as α-KG, Ac-CoA, and succinate) and key enzymes. This process forms the core mechanism of metabolic-epigenetic coupling. For instance, the pre-implantation stage of mammalian embryos undergoes a metabolic shift from maternal regulation to zygotic autonomous regulation, a critical transition closely linked to the dynamic restructuring of the TCA cycle at various stages.

Notably, key enzymes in the TCA cycle, such as pyruvate dehydrogenase complex (PDC), isocitrate dehydrogenase 2 (IDH2) and ATP citrate lyase (ACLY), undergo nuclear translocation at specific developmental stages. These enzymes create a localized metabolic microenvironment within the nucleus, directly catalyzing the production of high concentrations of epigenetic molecules: IDH2 nuclear translocation catalyzes the formation of α-KG, which regulates the activity of TET dioxygenase and influences DNA demethylation (Tahiliani et al., 2009). Meanwhile, citric acid from mitochondria is converted into Ac-CoA by ACLY within the nucleus, driving the critical histone acetylation (such as H3K27ac), promoting chromatin opening and ZGA (Wellen et al., 2009; Takahashi et al., 2006).

This review will provide an important theoretical framework for a deeper understanding of the metabolic etiology of developmental abnormalities and the development of new reproductive medicine intervention strategies (such as optimizing embryo culture medium and establishing an embryo quality assessment system with metabolic markers).

## 2 Metabolic bases of TCA cycle and energy demand of embryonic development

### 2.1 Energy metabolism characteristics in early embryonic development

In the early stages of embryonic development, two primary energy sources are present. First, the fertilized egg, being the largest cell in the human body, is rich in energy substances and can rely on the oocyte maternal metabolic reserves (Zhu and Wang, 2021). Second, early embryos can also obtain energy through exogenous metabolites, such as tubal fluid and uterine fluid (Leese, 2015; Lane and Gardner, 2000; Leese, 2003).

Embryonic metabolism serves a dual purpose: it maintains cellular stability and function through energy metabolism, and provides the raw materials for cell components and secreted substances through metabolite synthesis (Kaneko, 2016; Lu et al., 2021; Tippetts et al., 2023). Glucose, as a key carbon source, supports embryonic development through glycolysis and the TCA cycle (Leese, 2015; Lane and Gardner, 2000; Brown and Whittingham, 1991). Before the blastocyst stage, the embryos glucose metabolic activity is low, but during the blastocyst formation stage, its utilization efficiency significantly increases (Sharpley et al., 2021; Folmes et al., 2012). Isotope tracing experiments show that in the 2-cell stage, glucose carbon is primarily used for nucleotide ribosylation, while almost no glucose-derived markers are detected in the intermediates of the TCA cycle, indicating that early glucose functions are mainly biosynthetic rather than energy metabolic (Sharpley et al., 2021). It is also important to note that the energy sources for early embryonic development are not limited to glucose; they include lipids and other metabolites, forming an energy network (Tu et al., 2023; Zhang et al., 2024). As early as 2013, Chang et al. reviewed the findings that when embryonic stem cells (ESCs) differentiate, the level of glycolysis sharply decreases, while glucose and fatty acid-driven OXPHOS develops significantly (Shyh-Chang et al., 2013). Recent studies have further clarified the relationship between these processes, showing that glucose can overcome the 2-cell block in embryonic development by enhancing lipid synthesis (Wang et al., 2023).

In a low-oxygen environment of the fallopian tube (Fischer and Bavister, 1993; Varum et al., 2009), the embryos adaptation to pre-implantation hypoxia is crucial. Leese (1995) proposed that embryos use the Warburg effect (Vander Heiden et al., 2009; Warburg, 1956) to convert glucose into lactic acid, rapidly producing ATP and providing precursors for biosynthesis. This process may also promote implantation through local acidification (Leese, 1995; Zhou et al., 2012; Smith and Sturmey, 2013). Interestingly, pre-implantation embryos in humans and other mammals can still develop under hypoxic or OXPHOS inhibition conditions (Varum et al., 2009; Cho et al., 2006; Kondoh et al., 2007).

The regulation of ROS in pre-implantation embryos is crucial for their development. ROS, such as H_2_O_2_, regulate embryonic development through redox signaling at physiological concentrations, but pathological overproduction can lead to DNA damage and cell death (Sies and Jones, 2020). In mice, about 70% of the oxygen consumption during the cleavage stage is due to non-mitochondrial ROS production mediated by NADPH oxidase (Manes and Lai, 1995; Leese, 2012). Recent studies have shown that when mouse embryos are exposed to oxidative stress, increased ROS levels disrupt the dynamic balance of H3K4me3 and H3K27me3, leading to a decrease in the expression of key genes involved in ZGA (e.g., Hsp70.1 and Hsc70), which results in a 2-cell arrest (Ning et al., 2023).

### 2.2 Metabolic patterns shift from glycolysis to TCA cycle

In the early stages of mammalian embryogenesis, during the cleavage stage, the embryo primarily relies on glycolytic end products for energy. Within 24 h after fertilization, in mouse embryos, mitochondria are immature and round, with sparse cristae and low efficiency in OXPHOS (Cho et al., 2006; Oh et al., 2005; Ezashi et al., 2005). During this phase, glucose oxidation follows a dual-track pathway: after being oxidized to pyruvate, the majority is converted to lactate via lactate dehydrogenase (LDH), while only a small portion enters the mitochondria to participate in the TCA cycle. This contrasts sharply with the blastocyst stage (Lane and Gardner, 2000; Brown and Whittingham, 1991; Folmes et al., 2012; Miyazawa and Aulehla, 2018). Hayashi et al. conducted an intriguing experiment by introducing somatic mitochondria into mouse embryos at the 2-cell stage. At this stage, some mitochondria even transformed into round shapes, indicating that the mismatch between mitochondrial morphology and function disrupts NAD+/NADH homeostasis, leading to disordered ZGA timing and developmental arrest (Hayashi et al., 2025). It is also important to note that pyruvate is essential during the 1-2 cell stages; without pyruvate, the embryo cannot develop. After this stage, both pyruvate and lactic acid can promote development (Brown and Whittingham, 1991; Folmes et al., 2012; Lane and Gardner, 2005; Nagaraj et al., 2017).

**FIGURE 1:**
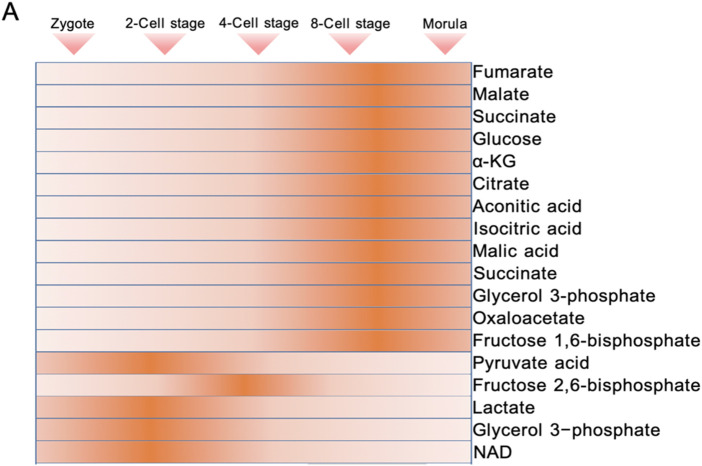
Dynamics of energy metabolic intermediate levels during preimplantation embryonic development.

The essence of metabolic mode conversion is the phased expression of genes related to glycolysis and TCA cycle (Figure 1). In mouse 8-cell embryos, the expression of TCA cycle enzymes and key metabolites (such as isocitrate, α-KG, succinate, malate) began to increase, and the circulating enzymes reached their peak in the blastocyst stage (Li J. et al., 2022; Zhao et al., 2021). Single cell sequencing data indicates that during the morula stage, human embryos show a significant upregulation of glycolytic genes such as SLC2A1 and LDHA. TCA cycle genes, including PDHB and CS, reach their peak levels during the blastocyst stage (Zhao et al., 2019). A similar pattern is observed in buffalo embryos: before the maternal regulation stage (MZT), glycolytic genes like HK and PFK are highly expressed, while after ZGA, oxidative phosphorylation genes such as PDH take the lead (Kumar et al., 2013). This suggests that TCA cycle activity begins to increase later in development.

## 3 Nuclear localization and developmental regulation of TCA cycle enzymes

### 3.1 Embryonic genome activation

During embryonic genome activation, MZT and ZGA are two closely linked core events in early embryonic development. MZT is the transitional stage where the embryo shifts from relying on maternal gene products (e.g., mRNAs, proteins) to being governed by the zygote’s own genome (Kojima et al., 2025). This process includes two key steps (Jukam et al., 2017).

First, degradation of maternal materials—clearing maternally accumulated transcripts and proteins in the oocyte (Jukam et al., 2017). In early embryonic development, MZT plays a critical role in regulating cell division progression and triggering zygotic gene activation, laying the foundation for cell differentiation and subsequent development. The primary goal of gene expression at this stage is to prepare the molecular conditions necessary for initiating gastrulation, a process involving complex cell movements and germ layer patterning (Kojima et al., 2025). It is through this phase that cells within the embryo begin to acquire distinct fates and form specific morphological structures (Jukam et al., 2017).

**FIGURE 2:**
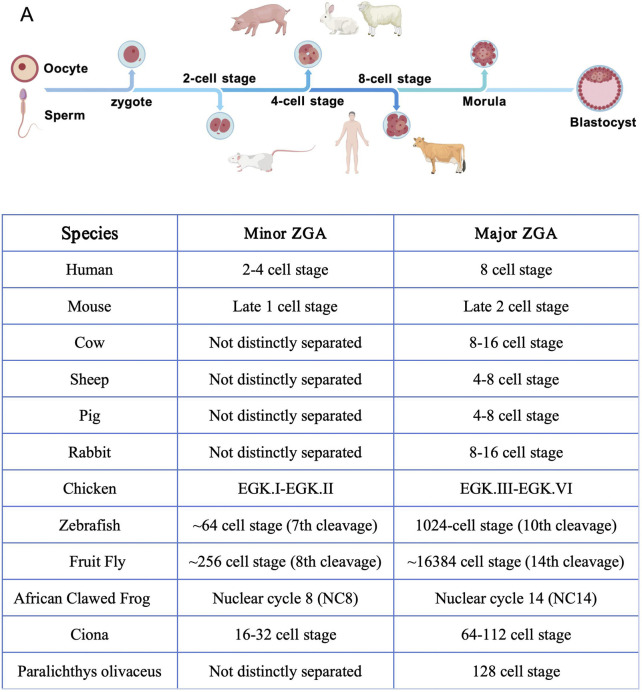
ZGA time series of species.

Second, ZGA occurs. ZGA, the starting point of autonomous embryonic development, is involved in the release from maternal control and the initiation of zygotic transcription (Zernicka-Goetz et al., 2009; Sozen et al., 2014). In mammals, ZGA is divided into two stages: major ZGA and minor ZGA (Schulz and Harrison, 2019; Zhang et al., 2025). Minor ZGA refers to the low-intensity, small-scale initial transcriptional activation of the genome occurring in early embryonic development, while major ZGA represents a large-scale, burst-like transcriptional activation event during embryonic development. For example, in mice, major ZGA occurs during the s phase of the fertilized egg and the G1 phase of the 2-cell stage, resulting in low-abundance, non-spliced initial transcripts (Abe et al., 2018; Jukam et al., 2017). Minor ZGA, on the other hand, occurs in the late 2 cell stage, driving large-scale gene expression and triggering the degradation of maternal mRNA (Eckersley-Maslin et al., 2018; Chen et al., 2023). There are also significant differences in the timing of ZGA across species (Schramm and Bavister, 1999; Christians et al., 1994):in humans, major ZGA waves occur during the 2–4 cell stage, while the primary wave occurs at the 8-cell stage; in cattle, ZGA is completed during the 8–16 cell stage (De Sousa et al., 1998; Frei et al., 1989; Li et al., 2025; Bu et al., 2022; Rengaraj et al., 2020; Wei et al., 2024; Hao et al., 2021) (Figure 2).

MZT is the “control handover” process in embryonic development, while ZGA marks the completion of this handover. Through spatiotemporal coupling of maternal degradation and zygotic activation, the two ensure a smooth transition of the embryo from gametic genetic information to zygote-autonomous development. Disruptions to the regulatory network (e.g., transcription factor abnormalities, epigenetic dysregulation) directly lead to ​developmental failure, while interventions targeting ZGA may provide a new pathway to enhance the success rate of assisted reproductive technology (ART).

### 3.2 Metabolic regulations of genome activation

In the ZGA process, DNA methylation plays a crucial role in the dynamic initiation of gene expression (Eckersley-Maslin et al., 2018). DNA methylation is a covalent modification of the fifth carbon atom (5 mC) of cytosine (Zhao et al., 2023), primarily occurring at CpG sites in DNA sequences. When these regions are densely present, they are known as CpG islands (CGIs), which are commonly found in the region of gene promoters. The regional remodeling of DNA methylation, in collaboration with transcription factors, activates the zygotic genome, regulating embryonic development and the reprogramming of gene expression (Eckersley-Maslin et al., 2018).

Histone acetylation acts as the key to unlocking the tightly packed chromatin structure during ZGA, leading to large-scale transcription of the genome. Histone acetylation neutralizes the positive charge of histones through HATs, reducing their electrostatic attraction to DNA. This process transforms compact heterochromatin into loose euchromatin, exposing gene promoter regions (such as the pluripotency genes Oct4 and Nanog), allowing transcription factors and RNA polymerases to bind (Ryall et al., 2015).

The dialogue between acetylation and methylation, representing fluctuations in metabolite levels such as α-KG and Ac-CoA, is crucial for the fate determination of embryonic development (Dai et al., 2020). Dahl et al. observed in 8-cell embryos that the transcription start site (TSS) region of the ZGA gene not only maintains the H3K4me3 modification but also shows a significant enrichment of H3K27ac (Dahl et al., 2016). This co-location pattern of H3K4me3 and H3K27ac suggests that DNA demethylation and histone acetylation may work together to create an epigenetic environment conducive to transcriptional activation through the coordinated supply of metabolic intermediates, such as nuclear Ac-CoA and α-KG.

### 3.3 The TCA cycle drives ZGA activation

The ZGA process requires a dynamic supply of metabolic products from the mitochondrial TCA cycle, with pyruvate playing a crucial role. In mouse embryos, if pyruvate is absent at the 2-cell stage, the embryo fails to complete spindle assembly and remains in the metaphase. Supplementing pyruvate can restore genomic activation, directly demonstrating its essential role in ZGA (Nagaraj et al., 2017; Whittingham and Biggers, 1967; Biggers et al., 1967; Biggers et al., 1965). Interestingly, only a small amount of pyruvate is fully oxidized to CO_2_ or converted into lactic acid in the embryo; most is transported through the mitochondrial membrane into the TCA cycle, where it generates key intermediates such as α-KG and Ac-CoA (Lane and Gardner, 2000). Recent studies by Li et al. further confirmed that when the pyruvate concentration in the culture medium drops to 20% of normal levels, mouse embryos not only stagnate at the 2-cell stage but also exhibit epigenetic disorders, including low methylation of the entire genome DNA, weakened histone methylation marks (H3K4me2/H3K9me2/H3K27me2), and abnormal increases in m6A RNA methylation (Li P. et al., 2022). In this process, the TCA cycle not only provides ATP for energy metabolism but also directly participates in epigenetic modification reprogramming through intermediate metabolites, driving chromatin remodeling and gene activation.

Early studies have shown that key enzymes of the TCA cycle exhibit dynamic localization in the nuclei of pre-implantation mammalian embryos, a phenomenon closely linked to the ZGA process. For instance, Nagaraj et al. observed that during ZGA in mouse embryos, enzymes such as PDH, CS, and ACO2 briefly enter the nucleus, accompanied by a transient peak of Ac-CoA and α-KG within the nucleus, which directly drives histone modification and DNA demethylation (Nagaraj et al., 2017). This phenomenon was also confirmed in the pyruvate deprivation experiment: embryos lacking PDH nuclear function, due to insufficient Ac-CoA, could not initiate histone acetylation, ultimately leading to ZGA failure and developmental arrest. The nuclear localization of these enzymes is strictly timed, their entry and exit from the nucleus are synchronized with the initiation and completion of ZGA, suggesting that their functions are directly linked to the epigenetic remodeling of the embryonic genome (Nagaraj et al., 2017).

**FIGURE 3:**
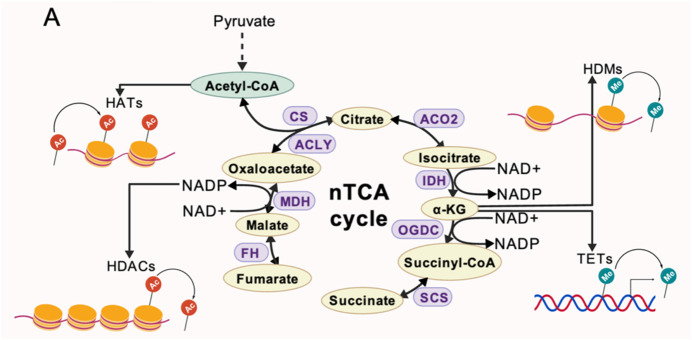
A non-classical TCA metabolic pathway localized within the nucleus, where key enzymes catalyze the generation of metabolites required for epigenetic modifications. Acetyl-CoA drives histone acetylation, while NAD^+^/NADP^+^ cofactors facilitate histone deacetylation. α-KG serves as a cofactor for demethylation enzymes (HDMs/TETs) to regulate histone/DNA methylation. SDH deficiency leads to succinate accumulation, triggering a reverse reaction catalyzed by FH/MDH2 to sustain the cycle operation.

According to traditional theory, intermediates of the TCA cycle must be actively transported from the mitochondria into the cell nucleus, but this process is insufficient to meet the immediate demands of chromatin remodeling during ZGA. The recently proposed *in situ* synthesis within the nucleus hypothesis suggests that nuclear-localized TCA cycle enzymes can directly catalyze the production of metabolites, creating a local high-concentration microenvironment (Liu et al., 2021; Kafkia et al., 2022; Li W. et al., 2022). For example, Kafkia et al. confirmed through isotope tracing and proximity labeling techniques that a subnetwork of the TCA cycle, consisting of enzymes such as ACO2, IDH3G, and OGDH, exists in mammalian cell nuclei, capable of directly using citric acid and glutamine to produce regulatory molecules like succinic acid/succinyl-CoA (Kafkia et al., 2022). Similarly, nuclear-localized PDH can directly catalyze the conversion of pyruvate to Ac-CoA, providing a readily available substrate for histone acetylation (Russo et al., 2024). This mode of operation of intranuclear metabolic pathways challenges the traditional view of mitochondrial centrality (Figure 3).

### 3.4 Nuclear translocation mechanism of TCA intermediate products

The nuclear translocation of key enzymes in the TCA cycle plays a crucial role in early embryonic development, involving cross-regional collaboration between mitochondria and the nucleus. Traditionally, small molecule intermediates of the TCA cycle, such as pyruvate,α-KG, and citrate, can enter the nucleus through passive diffusion or active transport via the nuclear pore complex (NPC) under the influence of nuclear localization signals (NLS) (Wente and Rout, 2010; Boon, 2021). For example, citrate is transported to the nucleus via the mitochondrial citrate carrier (CIC), also known as SLC25A1, where it is catalyzed by ACLY to form Ac-CoA, which directly drives histone acetylation and promotes chromatin opening (Williams and O'Neill, 2018; Arnold et al., 2022). However, large molecule intermediates or enzyme complexes, such as PDC and OGDC, are too large to be transported by the NPC, necessitating non-classical mechanisms. For instance, the giant enzyme complex composed of PDCE1-3 subunits can be transported from the mitochondrion to the nucleus in its complete form under proliferative stimulation, catalyzing the conversion of pyruvate to Ac-CoA, providing a substrate for histone acetylation within the nucleus (Sutendra et al., 2014; Chueh et al., 2011; Matsuda et al., 2016; Chen et al., 2018). The nuclear localization of these complex challenges traditional understanding, as they are much larger than the NPCs transport limit, and the subunits lack NLS.

PDC can enter the membrane contact sites (MCS) between mitochondria and the nuclear membrane (Campanella and Kannan, 2024). As early as 2009, Van Blerkom reviewed the significant perinuclear clustering and dynamic reconfiguration of mitochondria during early mammalian embryonic development. After fertilization, mitochondria form characteristic clusters around the male and female pronuclei and disperse in an orderly manner before the first cleavage. During subsequent embryonic divisions, they repeatedly exhibit a perinuclear clustering-dispersion cycle pattern that is cell cycle-dependent (Van Blerkom, 2009). Recent studies by Zervopoulos et al. in other cell types have shown that mitochondria form contact points with the nuclear membrane through dynamic anchoring mediated by mitochondrial fusion protein 2 (MFN2). The process is characterized by the rolling of mitochondria on the nuclear membrane surface followed by stable tethering, and then the PDC subunits penetrate the nuclear lamina through nuclear membrane endocytosis and enter the nucleoplasm. Key experimental evidence indicates that inhibiting MFN2 expression significantly reduces mitochondrial-nuclear membrane contacts and PDC nuclear localization, while blocking the function of the NPC has no effect on PDC nuclear enrichment, thereby confirming that this pathway has non-classical transport characteristics (Ning et al., 2023; Li W. et al., 2022; Zervopoulos et al., 2022). This mechanism is significantly associated with the observed mitochondrial dynamic reorganization in early mammalian embryos. Although direct evidence of MFN2 in the embryonic system is still lacking, the non-classical transport pathway characteristics suggest that PDC may achieve nuclear translocation in embryos through a conserved membrane contact mechanism.

The PDC complex enzyme may enter the nucleus through dissociation. Lee et al. (2020) found that PDC can dissociate into subunit complexes (such as E1α_2_β_2_ tetramers)at physiological salt concentrations (150 mM NaCl), and its enzymatic activity decreases with increasing salt concentration (Lee et al., 2020). This suggests that the metabolic plasticity of PDC supports its entry into the nucleus in subunit complex form, followed by reassembly into a functional complex within the low-ionic microenvironment of the nucleus. Sutendra et al. (2014) also supported this hypothesis through siRNA experiments: silencing PDC-E1 led to a reduction in nuclear localization of all subunits, indicating that PDC must function as a complete complex within the nucleus (Sutendra et al., 2014; Stacpoole and Dirain, 2024). After dissociation, PDC is sufficient to be exported from mitochondria via mitochondrial-derived vesicles (MDVs), but it cannot pass through the nuclear pore complex (e.g., E2p). Its final passage through the nuclear pore complex may still require interaction with HSP70/90 chaperones. During mammalian ZGA, enzymes such as PDH and CS may bind to HSP70/HSP90 through O-glycosylation modifications, forming a complex that enters the nucleus through glycosylated pores in the nuclear membrane, a process that does not depend on the classical NLS or NPC (Nagaraj et al., 2017). Functional studies have shown that inhibiting HSP90 (e.g., 17-AAG)blocks the nuclear localization of PDH and causes embryonic development to stall at the 2-cell stage (Nagaraj et al., 2017), while HSP70 is present in cells The increased expression level of S period is synchronous with the nuclear translocation of PDC, suggesting its dynamic regulation characteristics (Sutendra et al., 2014). According to current research, PDC still cannot pass through NPC, but the chaperone function of HSP70 may realize the transport of macromolecules through local nuclear membrane remodeling or pore expansion.

## 4 Regulatory functions of TCA intermediate products

### 4.1 Ac-CoA

Ac-CoA serves as the substrate donor for lysine acetyltransferase (KAT), playing a central role in epigenetic regulation. Its dynamic generation and metabolic reprogramming directly influence chromatin accessibility and gene expression patterns (Cai et al., 2011). KAT can be classified into two subtypes based on their subcellular localization: Type A (in the nucleus) and Type B (in the cytoplasm). based on their domain characteristics, sequence homology, and functional specificity, type A is further divided into five major families: GNAT, MYST, CBP/p300, transcription factor-related, and nuclear receptor co-activator (Shvedunova and Akhtar, 2022). KAT transfers Ac-CoA acetyl group to the ε-amino group of lysine residues in target proteins, such as histones or non-histones, forming an acetylation. The acetylation of histone H3 neutralizes the positive charge on lysine residues, reducing the interaction between DNA and histones, leading to an open chromatin conformation that regulates gene expression in stem cells (Cai et al., 2011). Ac-CoA can regulate major ZGA through histone H3K27ac (Zhang et al., 2025). In embryonic stem cells, active glycolysis promotes histone acetylation by maintaining high levels of Ac-CoA, driving the open expression of pluripotency genes. Conversely, inhibiting glycolysis or blocking the use of Ac-CoA precursors forces stem cells to differentiate prematurely (Moussaieff et al., 2015).

**FIGURE 4:**
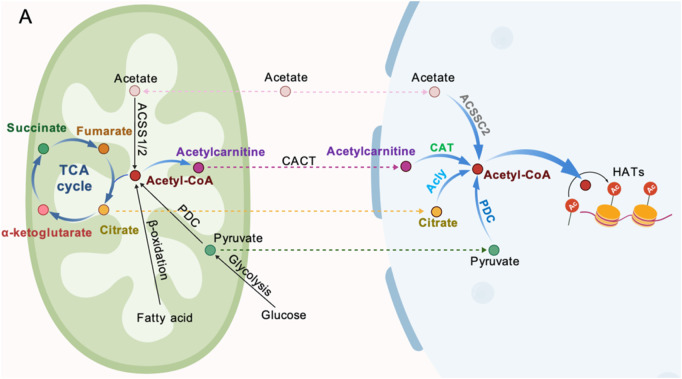
Sources of Ac-CoA generation. **(A)** Acetate can be converted to Ac-CoA by ACSSC2. **(B)** Mitochondria-derived acetylcarnitine (from Ac-CoA) can be transported into the nucleus by CACT and subsequently converted to Ac-CoA by CAT. **(C)** Citrate can be cleaved to Ac-CoA by ALCY. **(D)** Pyruvate can be metabolized to Ac-CoA inside the nucleus by a nuclear-localized PDC.

The nuclear supply of Ac-CoA is regulated by multiple pathways. The co-localization of ACLY and the PDC subunit in the nucleus creates a local microenvironment for Ac-CoA synthesis, directly supporting the acetylation of histone H2B, H3, and H4, which is crucial during the cell cycle (Sivanand et al., 2017). In early pig embryos, PDHA1 (PDH E1α subunit, which may have a nuclear localization sequence) is localized in the nucleus, catalyzing pyruvate to Ac-CoA, significantly increasing the levels of H3K9Ac and H3K27Ac. Targeted knockout of PDHA1 results in a reduction of acetylation by more than 60%, accompanied by a decrease in the expression of genes such as EIF1A, leading to embryonic arrest at the 4-cell stage. Overexpression of PDHA1 can partially restore acetylation levels and blastocyst formation rates, providing direct evidence for the nuclear synthesis of Ac-CoA and its epigenetic regulation (Zhou et al., 2020). Recent studies by have revealed that PDH and other ketone dehydrogenases can form a local microenvironment with high Ac-CoA concentrations in chromatin active regions (such as enhancers/promoters) through stable binding with the Mediator complex, thereby maximizing the catalytic efficiency of histone acetyltransferases (HATs). However, this process can be blocked by nitric oxide (NO), which S-nitrosates the E3 subunit of PDH, leading to reduced Ac-CoA production and impaired histone acetylation, suggesting that oxidative stress during embryonic development may interfere with the ZGA process through a similar mechanism (Russo et al., 2024). In addition to the ACLY and PDC pathways, there is also a mitochondrial-derived acylcarnitine pathway The carnitine acyltransferase (CACT) transports succinate to the nucleus, where it is catalyzed by the nuclear-localized carnitine acetyltransferase (CAT) to regenerate Ac-CoA, forming a third salvage pathway (Madiraju et al., 2009). This mechanism can lead to a significant decrease in histone acetylation levels in fibroblasts with carnitine deficiency or CACT defects, and a similar mechanism may also occur during embryonic development. Acetyl-CoA synthase 2 (ACSS2) produces Ac-CoA from CoA molecules from the cell exterior or through deacetylation reactions catalyzed by lysine deacetylation enzymes (Russo et al., 2024; Stacpoole and Dirain, 2024). Additionally, studies have shown that signaling pathways can influence Ac-CoA production. For example, when mitochondrial activity is inhibited or glucose is scarce, the Akt signaling pathway enhances its enzymatic activity by phosphorylating ACLY at the Ser455 site, maintaining Ac-CoA levels within the nucleus (Lee et al., 2014). Meanwhile, AMPK phosphorylates ACSS2, promoting its nuclear translocation and using the acetic acid released from histone deacetylation to re-synthesize Ac-CoA, thus maintaining local histone acetylation levels (Boon, 2021; Moussaieff et al., 2015; Li et al., 2017; Mews et al., 2019). This metabolic adaptation is crucial for the survival of embryos in hypoxic or nutrient-limited environments (Figure 4).

### 4.2 α-KG

As a core metabolite in the TCA cycle, α-KG integrates energy metabolism and epigenetic regulation networks, playing a multifaceted role in cell proliferation and embryonic development. Its functions extend beyond ATP production and biosynthetic precursors; it also serves as a crucial cofactor forα-ketoglutarate-dependent dioxygenase, dynamically shaping chromatin state (Carey et al., 2015). In early mammalian embryonic development, α-KG regulates ZGA through two mechanisms. As a cofactor of TET enzymes, α-KG drives the active demethylation of paternal genomes, thereby relieving transcriptional repression. Additionally, it specifically erases histone methylation modifications via the JmjC domain of JHDM, reshaping the chromatin openness of promoter regions (Tsukada et al., 2006). Studies have shown that the spatiotemporal-specific erasure of H3K4me3 in mouse 2-cell embryos is essential for the precise activation of ZGA genes. Knocking out KDM5A/B results in ZGA gene silencing and embryonic developmental arrest (Dahl et al., 2016). In embryonic stem cells, α-KG maintains a high α-KG/oxaloacetate ratio, promoting TET enzymes to oxidize 5mC to 5-hydroxymethylcytosine (5hmC), while activating JHDM to clear inhibitory histone modifications, thus maintaining the open chromatin state of pluripotency genes (Ispada et al., 2020). Supplementing cells with permeable α-KG significantly enhances the active erasure of histone H3K27me3, inhibiting the silencing of differentiation-related genes (Carey et al., 2015; TeSlaa et al., 2016).

**FIGURE 5:**
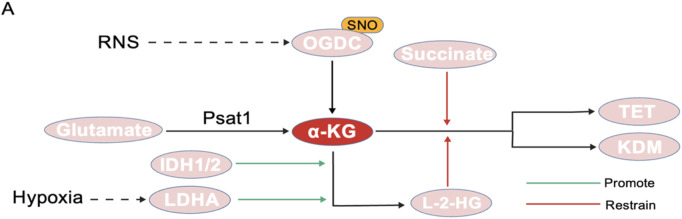
Generation and regulation of α-KG. **(A)** RNS can inhibit OGDC activity through S-nitrosylation. **(B)** Psat1 catalyzes the oxidative deamination of glutamate to generate α-KG. **(C)** IDH1/2 and LDHA can promote the conversion of α-KG to L-2-HG. **(D)** L-2-HG and succinate can competitively inhibit α-KG, thereby reducing its binding to TET and KDM. ​​

α-KG is primarily produced by the key rate-limiting enzyme IDH in the TCA cycle. Chromatin modification factor EP400 influences the nuclear synthesis of α-KG by directly regulating the expression of IDH family genes, such as IDH3b. This regulation drives the dynamic erasure of H3K27me3 modifications and ensures the precise timing of ZGA (Tian et al., 2024). Some amino acids, like arginine and glutamine, also support the production of α-KG (Carey et al., 2015; Dumollard et al., 2009; Kaelin and McKnight, 2013). Recent studies have shown that reactive nitrogen species (RNS) can inhibit the activity of the α-ketoglutarate dehydrogenase complex (OGDC) through S-nitrosylation of its thiolate arm, thereby reducing α-KG production (Seim et al., 2023). Post-translational modifications of this metabolic enzyme may indirectly interfere with the DNA demethylation process mediated by TET enzymes by lowering the ratio of α-KG to succinate. Additionally, phosphoseryl aminotransferase (Psat1), a direct target gene of Oct4/Sox2/Nanog (OSN), regulates the cellular level of α-KG by catalyzing the oxidative deamination of glutamate. Its expression rapidly decreases during the 2-cell stage and early embryonic stem cell differentiation, leading to fluctuations in the nuclear level of α-KG (Tian et al., 2024; Hwang et al., 2016) (Figure 5).

The regulatory function of α-KG is closely linked to its metabolic antagonist, 2-hydroxyglutaric acid (2-HG), which maintains a dynamic equilibrium (Miyazawa and Aulehla, 2018). 2-HG consists of two isomers: L-2-HG and D-2-DG, both of which influence the de-methylation process in cells by affecting demethylase enzymes (Martínez-Reyes and Chandel, 2020; Penn et al., 2023). Research has shown that L-2-HG exhibits unique spatiotemporal distribution characteristics during early mammalian embryonic development: its levels significantly increase in oocytes and two-cell embryos after fertilization, but decrease as the embryo develops to the blastocyst stage. This change contrasts sharply with the gradual accumulation of α-KG, resulting in a significantly higher L-2-HG/α-KG ratio in the early stages of embryonic development compared to later stages (Zhao et al., 2021). L-2-HG acts as a competitive inhibitor of α-KG-dependent dioxygenase, blocking its catalytic function by occupying the enzymes active site, thereby interfering with DNA demethylation and histone modification erasure processes (Xu et al., 2011; Lu et al., 2012). Exogenous supplementation of L-2-HG has been experimentally confirmed to significantly inhibit the removal of histone methylation marks such as H3K4me3 and H3K9me3 during embryonic development, leading to a decrease in blastocyst formation rates and morphological abnormalities (Zhao et al., 2021). A study on tumors has proposed a new perspective: under hypoxic conditions, LDHA can selectively generate L-2-HG by reducing α-KG, which significantly increases H3K9me3 levels by inhibiting KDM4C and other histone demethylases (Chowdhury et al., 2011). This is consistent with the early hypoxic environment of embryos is similar, which can serve as a new idea to produce L-2-HG. Secondly, the acidic environment of early embryos can also be used as an influencing factor to stimulate the production of L-2-HG (Nadtochiy et al., 2016; Intlekofer et al., 2017). Although no studies have been conducted on the influence of the early acidic environment of embryos, this is also an entry point.

### 4.3 NAD+/NADH

The NAD+/NADH ratio acts as a regulator of cellular energy synthesis, and its homeostasis can lead to developmental disorders in embryos. The reductive equivalents NADH produced by the TCA cycle are oxidized by the, ETC complex to form NAD+, which provides energy for OXPHOS (Birsoy et al., 2015; Nicholls and Budd, 2000; Pagliarini and Rutter, 2013). Recent studies have shown that in mouse 2-cell embryos, the level of NAD + decreases by about 70% compared to the 1-cell stage, while the level of NADH only slightly decreases, leading to a significant reduction in the total NAD(H) pool (Sharpley et al., 2021; Li J. et al., 2022). This imbalance in the NAD+/NADH ratio inhibits PDH activity, forcing the embryo to rely on LDH to maintain metabolic homeostasis (Sharpley et al., 2021; Luengo et al., 2021; Titov et al., 2016). The Sharpley team further demonstrated that exogenous supplementation of NAD + can partially reverse the developmental arrest at the 2-cell stage caused by pyruvate deficiency (Sharpley et al., 2021). Alba Luengo and colleagues proposed a hypothesis that pyruvate oxidation requires NAD + as a coenzyme, whereas conversion to lactic acid does not. Therefore, when cells need more NAD + for oxidation (such as biosynthesis or antioxidant activities), pyruvate is forced to convert to lactic acid (Luengo et al., 2021). This explains the Warburg effect from the perspective of the NAD+/NADH ratio.

The Sirtuin family is a group of NAD + -dependent deacetylases that remove acetyl, succinyl, propionyl, and other acetylation from proteins, playing a crucial role in the regulation of stem cells (Cantó et al., 2015). The NAD+/NADH balance undergoes significant fluctuations early after fertilization. By regulating the activity of the SIRT1 deacetylase, it influences the dynamic erasure of the H3K27ac modification (Papagiannakis et al., 2017). When the embryo develops to the late two-cell stage, SIRT1 catalyzes the deacetylation of H3K27ac in the promoter region of the major ZGA gene by binding to NAD+, preventing the delayed over-activation of the major ZGA gene in the late two-cell embryo, thus ensuring the normal development of the embryo (Li J. et al., 2022). Furthermore, recent studies have shown that SIRT1 maintains chromatin stability by deacetylating H4K16 in the pronucleus of the fertilized egg. The maternal SIRT1 protein is recruited into the nucleus during zygote formation through a non-translated mechanism, which is essential for the early embryo to overcome developmental delays (Nevoral et al., 2024).

The association between NAD + metabolism and stem cell fate is also evident in its direct regulation of epigenetic modifications. The level of NAD+ is regulated by Nicotinamide Phosphoribosyltransferase (NAMPT), which acts as the rate-limiting enzyme in the NAD + salvage pathway (Li J. et al., 2022; Berger et al., 2005; Cambronne et al., 2016). Under conditions of DNA damage or nutritional stress, NAMPT activity is upregulated to enhance NAD + synthesis and maintain Sirtuins function. Studies have shown that inhibiting NAMPT can lead to embryonic development being arrested at the four-cell stage, with an abnormal increase in H3K27ac levels in the late two-cell stage (Li J. et al., 2022). Additionally, PARP1, a NAD + -consuming enzyme, continuously depletes NAD + under physiological conditions. Its inhibitor, FK866, can increase NAD + levels and improve mitochondrial function. However, excessive supplementation of Nicotinamide Mononucleotide (NMN) exacerbates the H3K27ac erasure barrier, suggesting that NAD + metabolism has a precise regulatory threshold (Streffer et al., 1974).

## 4 Succinic acid

Succinate, a key intermediate in the TCA cycle, forms a deep regulatory network with the HIF-1α signaling pathway through a pseudo-hypoxia mechanism, coordinating the reprogramming of embryonic metabolism and epigenetic remodeling. In the early stages of embryonic development, the low-oxygen environment in the mothers uterus inhibits SDH activity, leading to succinate accumulation (Koivunen et al., 2012). Succinate competitively inhibits PHD, blocking the ubiquitination degradation of HIF-1α, stabilizing it and activating downstream target genes, which drives the shift from glycolysis to support energy supply. For example, in mouse blastocysts with SDH defects, increased succinate levels enhance the expression of HIF-1αtarget genes, improving embryo survival rates; whereas embryos with HIF-1α knock out, due to inhibited glycolysis, experience developmental arrest (Iyer et al., 1998; Ryan et al., 1998), demonstrating the central role of the succinate-HIF-1αaxis in metabolic adaptation.

Functional gain mutations in IDH1/2 not only impair their normal catalytic functions but also lead to the abnormal conversion of α-KG into 2-HG. In mutant cells, the concentration of 2-HG can rise to millimolar levels (Xu et al., 2011; Chowdhury et al., 2011; Ward and Thompson, 2012; Losman and Kaelin, 2013; Ward et al., 2010). Additionally, IDH mutations reduce NADPH synthesis, weakening the embryo ability to clear ROS and exacerbating mitochondrial oxidative stress, ultimately triggering p53-dependent apoptosis (Chowdhury et al., 2011). Recent studies in pig embryos have shown that the depletion of α-KG caused by IDH2/GLUD1 knockdown results in abnormally high methylation of H4K20me3 at the 4-cell stage, inhibiting the expression of genes involved in ZGA. However, exogenous supplementation of α-KG or knockout of the methyltransferase KMT5C can partially restore the developmental process (Zhan et al., 2024). This suggests that IDH2 mutations may affect ZGA by depleting α-KG.

Succinate regulates DNA methylation and histone modification dynamics by interfering with α-KG-dependent epigenetic enzymes. As a competitive inhibitor of TET and KDMs (Chandel et al., 2016; Mahmoud, 2023), the accumulation of succinate leads to high DNA methylation and enhanced H3K27me3 modification, which inhibits the differentiation of primordial germ cells (PGCs) and the activation of pluripotency genes (Xiao et al., 2012). Studies have shown that reducing the ratio of α-KG to succinate can inhibit the activity of TET and JMJD3, leading to abnormal accumulation of H3K27me3 and DNA methylation, thereby hindering the lineage differentiation of embryonic stem cells (Carey et al., 2015; TeSlaa et al., 2016).

The lysine succinylation mediated by succinyl-CoA regulates the activity of metabolic enzymes and chromatin status by altering protein charge and conformation. Wangs team demonstrated that OGDC achieves nuclear translocation through the nuclear localization signal (Arg224/Lys226) of the DLST subunit, forming a functional complex with histone acetyltransferase KAT2A. Crystal structure analysis revealed that the flexible loop3 region of KAT2A, particularly the Tyr645 residue, specifically recognizes succinyl-CoA and catalyzes the succinylation of the H3K79 site on histones (Wang et al., 2017). Recent studies indicate that mitochondrial-derived succinate primarily reduces the ratio of α-KG to succinate in the nucleus by inhibiting cytoplasmic OGDC activity, while nuclear-localized OGDC maintains local succinyl-CoA production through metabolic compartmentalization: In human embryonic stem cells, the nuclear-to-cytoplasmic distribution of OGDC exhibits G1/S phase-dependent fluctuations, and its enrichment in the nucleus is positively correlated with the level of H3K79 succinylation (Kafkia et al., 2022).

## 5 Discussion

This review systematically elucidates the core regulatory role of the TCA cycle in early embryonic development, which goes beyond mere energy metabolism. It reveals a sophisticated regulatory network that involves dynamic metabolic reprogramming, the construction of a nuclear metabolic microenvironment, and precise epigenetic remodeling. This not only challenges the traditional view of the TCA cycle as merely an energy factory but also establishes its central role in developmental processes——through key intermediate metabolites (such as Ac-CoA, α-KG, and succinyl-CoA)and enzymes crucial for their nuclear localization (such as PDH, IDH, ACLY, and OGDC), the TCA cycle directly interacts with the epigenetic machinery, serving as a pivotal molecular hub that links cellular metabolic state to gene expression programs.

The core significance of the “metabolic-epigenetic coupling” mechanism lies in its role in ensuring the spatiotemporal specificity of cell fate determination. Embryonic development, particularly the transition from maternal control to zygotic autonomy (marked by ZGA), is a highly coordinated and irreversible process. The dynamic fluctuations of TCA cycle metabolites, such as the peak levels of Ac-CoA in the nucleus before ZGA and the phased changes in the α-KG/succinate ratio, along with the local high-concentration microenvironment they create in the nucleus, provide precise substrates and cofactors for histone modifications (acetylation, methylation, succinylation) and DNA methylation/de-methylation. This mechanism ensures that chromatin can be precisely remodeled at specific time windows (such as late 2-cell stage) and specific genomic regions (such as the promoters/enhancers of key ZGA genes), driving necessary gene activation or silencing. For example, the explosive production of Ac-CoA in the nucleus, catalyzed by PDH/ACLY, drives the enrichment of active marks like H3K27ac, which is the essential “key” to open chromatin and initiate major ZGA. Meanwhile, the level of α-KG, regulated by IDH/OGDC, precisely controls the activity of TET and JHDM, erases inhibitory marks (such as H3K27me3, H3K9me3), and promotes active DNA demethylation, clearing the way for the “awakening” of the zygotic genome. Additionally, mitochondrial-nuclear signaling (such as contact mediated by MFN2 and MDV transport) and the translation of metabolic enzymes are also involved Post-modifications (such as NO inhibition of PDH) provide an additional level of regulation and flexibility to respond to external stimuli (such as oxidative stress, nutrition). It is noteworthy that the nuclear metabolic microenvironment mechanism revealed in this study—specifically, the local metabolism-epigenetic coupling driven by nuclear localization of TCA cycle enzymes—may exhibit cross-system generalizability. In cancer, the α-KG/2-HG imbalance caused by IDH mutations triggers malignant epigenetic remodeling by inhibiting TET enzymes; whereas in iPSC reprogramming, exogenous α-KG can mimic embryonic metabolic pulses to enhance efficiency. This suggests that such a mechanism might represent a universal paradigm for cell fate regulation. Future studies need to further dissect its tissue-specific thresholds.

The current limitations of the research lie in although metabolic intermediates (such as α-ketoglutarate, S-adenosylmethionine, acetyl-CoA, etc.), by regulating the activity of epigenetic modification factors like DNA methyltransferases (DNMTs), histone deacetylases (HDACs), and TET dioxygenases, have been extensively reported in cell fate determination and gene expression reprogramming during early embryonic development, research on the reverse regulatory mechanism—that is, the feedback regulation of epigenetic modifications on metabolic pathways and metabolite synthesis—remains relatively weak in the field of embryonic development. Secondly, current research on the functions of metabolites within the cell nucleus is mainly limited to mouse models. In human embryos, only the nuclear localization of specific metabolites has been observed, and the specific biological functions and regulatory mechanisms of these metabolites have not yet been deeply explored.

The key challenge for future research is to elucidate the ultimate spatiotemporal precision of this network: how do specific metabolites precisely accumulate in specific genomic regions at specific developmental stages? What are the mechanisms of assembly, activity regulation, and dynamic interactions between nuclear TCA enzyme complexes and chromatin remodeling complexes (such as Mediator)? Are there developmental stage-specific metabolic checkpoints, and what are their thresholds? Exploring these questions will not only deepen our understanding of the regulatory logic at the dawn of life but also drive innovations in reproductive and regenerative medicine through metabolic interventions.
